# Effect of breast milk with or without bacteria on infant gut microbiota

**DOI:** 10.1186/s12884-022-04930-6

**Published:** 2022-07-26

**Authors:** Ting Huang, Zichun Zeng, Xinyuan Liang, Xiaomei Tang, Huijuan Luo, Dongju Wang, Juan Zhou, Xiaomin Xiao

**Affiliations:** 1 Department of Obstetrics and Gynecology, The First Affiliated Hospital of Jinan University, Guangzhou, China; 2 Department of Obstetrics and Gynecology, Chengdu Fifth People’s Hospital, Chengdu, China; 3 Department of Obstetrics, The Second Clinical Medical College, Jinan University (Shenzhen People’s Hospital), Guangzhou, China

**Keywords:** Breastfeeding, Human milk, Infant, Gut microbiota

## Abstract

**Background:**

The breast milk microbiome could be a source of infant intestinal microbiota. Several studies have found that some breast milk is extremely low in bacteria or is even sterile. There are limited studies on the effect of milk without bacteria on the infant gut microbiota. The purpose of this study was to investigate the gut microbiota of infants fed with bacterial milk or sterile milk. Meanwhile, we attempted to find the cause of undetectable bacteria in milk.

**Methods:**

A total of 17 healthy pregnant women and 17 infants were enrolled in this study. Fecal samples were collected from full-term pregnant women. Milk samples and infant fecal samples were collected on the 14th postnatal day. Breast milk and fecal samples were examined using 16S rRNA sequencing technology. Pregnant women and infants were grouped according to milk with or without bacteria. To compare the differences in gut microbiota and clinical characteristics between groups.

**Results:**

Bacteria were detected in 11 breast milk samples, and the bacterial detection rate was 64.7%. Infants fed with bacterial milk showed higher Shannon index and Simpson index (*P* = 0.020, *P* = 0.048), and their relative abundance of *Lachnospirales*, *Lachnospiraceae* and *Eggerthellaceae* was markedly higher. In addition, there were more bacterial associations in the co-occurrence network of infants fed with bacterial milk. Pregnant women with sterile and bacterial breast milk showed no significant differences in their clinical characteristics, and microbial composition and diversity.

**Conclusions:**

Some breast milk from healthy postpartum women failed to be sequenced due to low microbial DNA quantities or is sterile. Research is needed to explore the reasons for this phenomenon. Infants fed with bacterial milk had higher Alpha diversity and more complex microbiota networks. These findings provide novel insight into milk microbiota and infant gut microbiota.

**Supplementary Information:**

The online version contains supplementary material available at 10.1186/s12884-022-04930-6.

## Background

Human milk provides the best nutrition for infants in early life. The sugar, lipids, proteins, macronutrients, and micronutrients in breast milk provide rich nourishment for infants. With the development of high-throughput sequencing technology, it was discovered that microorganisms are also present in human breast milk. Milk microbes can colonize the infant gut, affecting the establishment and development of the gut microbiome [1]. Early development of the infant gut microbiome is related to allergic disorders, atopic dermatitis, and asthma [2–5]. As a result, breast milk plays a critical role in the health of infants and the colonization of the intestinal microbial community.

In our previous study, 11 out of 25 milk samples from healthy pregnant women failed to be sequenced due to an insufficient quantity of bacteria [6]. This phenomenon was also observed in other studies [7–13]. It indicated that not all healthy lactating women’s milk contains microbiota. Currently, it is unclear what causes this phenomenon and whether infants fed with sterile milk have different microbial communities. Most studies on infant intestinal microbiota have focused on the effects of delivery modes [14], feeding modes [15], bioactive components of milk [16], and maternal diet [17, 18]. Limited data are available on the comparison of gut microbiota in infants fed with bacterial milk and sterile milk. A number of studies reported that delivery mode, geographical location, postpartum period, gestational age, feeding methods, BMI, and infant gender may cause individual differences in the composition of milk microbiota [12, 19–22]. In addition, it has been suggested that there may be an entero-mammary pathway in the human body, whereby dendritic cells were able to open tight junctions between intestinal epithelial cells and transported some bacteria from the intestine to the breast [23, 24]. This bacterial translocation occurs more frequently in women during late pregnancy and lactation [25]. Therefore, we speculated that gut microbiota may influence the construction of breast milk microbiota.

In this study, we detected the microbial communities from mother-neonate pairs’ feces and maternal milk. Our goal was to investigate whether bacterial milk and sterile milk have different effects on infant gut microbiota and whether gut microbiota influences the construction of the milk microbiota.

## Material and methods

### Participants

The participants in this observational study were 20–35 years old women who achieved singleton pregnancy spontaneously. Furthermore, women with pregnancy complications, infectious diseases or chronic diseases, as well as women who had used antibiotics or probiotics during pregnancy were excluded from the study. According to the above criteria, 23 pregnant women from the First Affiliated Hospital of Jinan University (Guangzhou, China) were enrolled in our study. All of the pregnant women delivered vaginally, and their infants’ Apgar scores were all normal. Meanwhile, their infants were also included in this study. All pregnant women who participated in the study signed an informed consent form.

### Collection of sample and clinical data

Fecal samples were taken from pregnant women after full term of pregnancy. On the 14th postnatal day, breast milk and infant feces were collected. Fecal samples were collected by professionals using sterile spoons and sterile boxes. Before collecting the milk, the mothers cleaned the nipple and surrounding skin with soap and water. Women wore sterile gloves to squeeze out the milk and filled in sterile tubes. All samples were transported on ice and then stored in the refrigerator at -80℃ until DNA extraction. We also collected clinical information about pregnant women and infants, such as maternal age, height, weight, gestational weeks, neonatal length, weight, and gender.

### DNA Extraction and sequencing

The CTAB/SDS technique was used to extract total bacterial DNA from samples. DNA concentration and purity were monitored on a 1% agarose gel. According to the concentration, DNA was diluted to 1 ng/μL with sterile water. The V3-V4 hypervariable region of 16S rRNA gene was amplified using a specific primer (341F-806R) with the barcode. All PCR reactions were performed with Phusion® High-Fidelity PCR Master Mix (New England Biolabs). The PCR products were mixed with the same volume of 1X loading buffer (containing SYB Green) and electrophoresed on a 2 percent agarose gel. PCR products were mixed in equal density ratio. The mixed PCR products were then purified using Qiagen Gel Extraction Kit (Qiagen, Germany). Sequencing libraries were generated by using TruSeq® DNA PCR-Free Sample Preparation Kit. The library was sequenced on a NovaSeq6000 platform after assessing on the Qubit@ 2.0 Fluorometer.

### OTU cluster and Species annotation

Each read was stripped of the barcode and the primer, and then they were spliced by using FLASH (V1.2.7, http://ccb.jhu.edu/software/FLASH/) [26]. Next, effective tags were generated by intercepting, filtering, and removing chimeric sequences from the raw tags. The Uparse algorithm (Uparse v7.0.1001,http://www.drive5.com/uparse/) was used to cluster all effective tags into sequences, and then all sequences were clustered into operational taxonomic units (OTUs) with 97% identity. Finally, representative sequences from each OTU were aligned to the SSUrRNA database (http://www.arb-silva.de/).

### Statistical and microbial analysis

Alpha diversity was used to assess diversity within samples, whereas Beta diversity was used to compare the composition of microbial communities between samples. The Simpson index and Shannon index were calculated in QIIME software (Version 1.7.0) to estimate Alpha diversity. A higher Shannon and Simpson index implies a community with greater diversity. Comparison of Alpha diversity indices between groups using the Wilcoxon test with GraphPad Prism software (Version 8.0.2). Beta diversity was demonstrated using Principal Coordinates Analysis (PCoA) based on weighted Unifrac distances. To estimate the correlation between genera, we calculated the Spearman coefficients for the top 100 genera by relative abundance. Genera with absolute Spearman correlation coefficients greater than 0.6 and *P* values less than 0.05 were considered for construction of the network. The visualization of each network and calculation of topological properties were performed using Gephi software (Version 0.9.2). The relative abundance histogram and the Venn diagram were plotted using R software. To further investigate biomarkers between different groups, we conducted linear discriminant analysis effect size (LEfSe). The clinical data were compared between groups using independent-samples t test or Chi-square test. A *P* value less than 0.05 was considered statistically significant.

## Results

### Sample sequencing results and participant characteristics

**Fig. 1:**
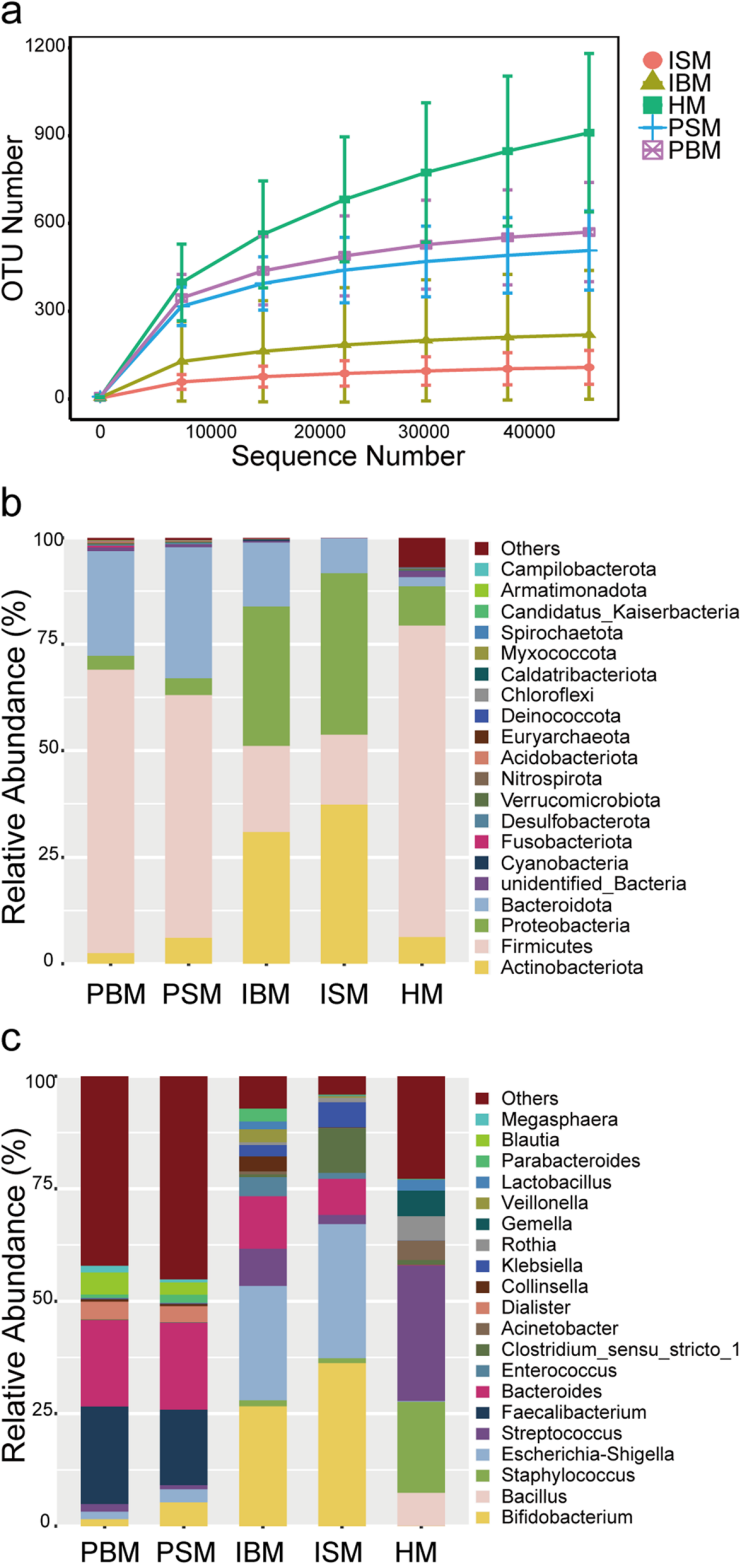
The rarefaction curve and relative abundance bar plot for each group. **a** Rarefaction curve. **b** Relative abundances of the top 20 taxonomy at the phylum level for each group. **c** Relative abundances of the top 20 taxonomy at the genus level for each group. PBM = Pregnant women with bacterial milk; PSM = Pregnant women with sterile milk; IBM = Infants fed with bacterial milk; ISM = Infants fed with sterile milk; HM = Human milk

Seventeen of 23 mother-infant pairs were eventually enrolled in our study. Five pairs were eliminated due to lack of breast milk or personal reasons, and one pair was excluded because the mother suffered from mastitis. Six of the 17 milk samples could not be sequenced due to low bacterial loads or the absence of bacteria. The detection rate of microbe in milk was 64.7%. Breast milk that failed to be sequenced in this study was tentatively considered sterile milk. Based on the sequencing results of breast milk, the subjects were divided into four groups: pregnant women with bacterial milk (PBM, *n* = 11), pregnant women with sterile milk (PSM, *n* = 6), infants fed with bacterial milk (IBM, *n* = 11), infants fed with sterile milk (ISM, *n* = 6). The rarefaction curves of samples in each group tended to be flat as the sequence number increased, indicating that the sequencing depth was sufficient (Fig. 1a).

There were no significant differences in age, height, weight, BMI, and gestational week between pregnant women, and there were no significant differences in birth weight, length, and gender between infants (Table 1).

**Table 1:** Characteristics of participants

	Bacterial milk	Sterile milk	*P*
Maternal characteristics			
Number of women	11	6	
Maternal age (year)	26.6 ± 1.91	26.8 ± 1.60	0.834
Height (m)	1.6 ± 0.59	1.6 ± 0.06	0.788
Gestational weight (kg)	64.6 ± 8.45	62.5 ± 5.81	0.599
Gestational BMI (kg/m^2^)	25.0 ± 2.52	24.7 ± 3.45	0.804
Gestational week (weeks)	39.5 ± 0.96	38.9 ± 1.17	0.232
Infant characteristics			
Number of infants	11	6	
Birth weight (g)	3200.0 ± 246.98	3083.3 ± 320.42	0.414
Birth body length (cm)	49.5 ± 1.04	49.0 ± 1.67	0.496
Gender (%)			0.627
Male	54.5	66.7	
Female	45.5	33.3	

### Microbiota profile of breast milk

The microbial composition of maternal breast milk was shown in Fig. 1b-c and Table 2. Among the microbial communities of breast milk, *Firmicutes* predominated (73.1%), followed by *Proteobacteria* (9.2%), *Actinobacteria* (6.3%), and *Bacteroidetes* (2.2%), while the relative abundance of the rest of the phylum was less than 1%. At the genus level, eight genera had mean relative abundances greater than 1%. They were *Streptococcus* (30.1%), *Staphylococcus* (20.2%), *Bacillus* (7.4%), *Gemella* (5.7%), *Rothia* (5.4%), *Acinetobacter* (4.3%), *Lactobacillus* (2.5%), and *Clostridium_sensu_stricto_1* (1.1%). Among the above genera, 73.1% of the bacteria were aerobic or facultative anaerobes, and 3.6% were anaerobes (Table 3).

**Table 2:** Relative abundances of the top four taxonomy at the phylum level for each group (%)

	HM	PBM	PSM	IBM	ISM
*Firmicutes*	73.1	66.5	57.0	20.2	16.4
*Proteobacteria*	9.2	3.3	4.0	32.8	38.0
*Actinobacteria*	6.3	2.5	6.1	31.0	37.4
*Bacteroidetes*	2.2	24.6	30.8	15.0	8.2

*HM* Human milk, *PBM* Pregnant women with bacterial milk, *PSM* Pregnant women with sterile milk, *IBM* Infants fed with bacterial milk, *ISM* Infants fed with sterile milk

**Table 3:** Milk bacteria with relative abundance greater than 1% and their relationship with oxygen

	Relative abundance (%)	Relationship with oxygen
*Streptococcus*	30.1	Facultative anaerobic
*Staphylococcus*	20.2	Facultative anaerobic
*Bacillus*	7.4	Aerobic or facultative anaerobic
*Gemella*	5.7	Aerobic or facultative anaerobic
*Rothia*	5.4	Aerobic
*Acinetobacter*	4.3	Obligate aerobic
*Lactobacillus*	2.5	Anaerobic
*Clostridium_sensu_stricto_1*	1.1	Strict anaerobic
*Other*	23.3	-

The relationship between genera and oxygen was obtained by searching the eighth edition of Berger’s Handbook of Bacteria

It was noted that specific gut bacteria such as *Bifidobacterium* and *Bacteroidetes* were present in 10 out of 11 milk samples. The presence of *Bifidobacterium* and *Bacteroides* was 90.9%, but their relative abundances were less than 1.0% (Supplementary Table S1).

### The influence of milk microbiota on infant gut microbiota

**Fig. 2:**
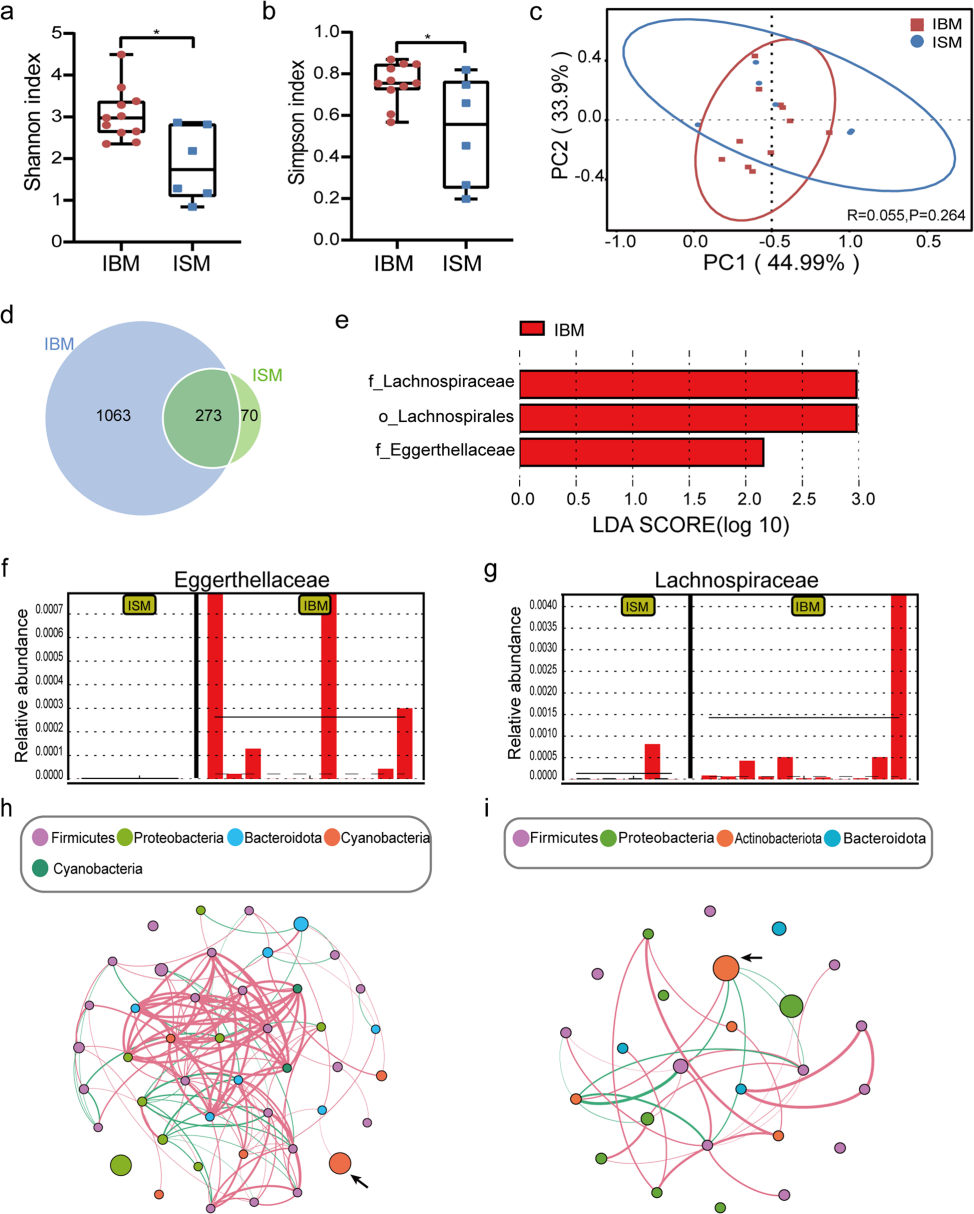
Microbial analysis of infant fecal samples. Alpha diversity estimated by Shannon indices (**a**) and Simpson indices (**b**) showed significant differences in groups. **c** PCoA based on weighted Unifrac distances indicated there is no differences in groups. Statistical significance was calculated by the Anosim test. **d** Venn diagram demonstrated that the IBM group had more unique OTUs. **e** LEfSe analysis compared all taxonomy between groups. Identified three biomarkers when LDA score = 2. **f** and **g** showed the relative abundance of *Eggerthellaceae* and *Lachnospiraceae* in each sample, respectively. Co-occurrence networks of gut microbiota at the genus level were showed in (**h**) and (**i)**. **h** Co-occurrence network of the IBM group. **i** Co-occurrence network of the ISM group. Nodes with relative abundance less than 0.005% were removed. The nodes were colored by phylum, and nodes’ size was proportional to the relative abundance of genera. Edges represented the relationships between the nodes. A positive correlation was shown by red edges, while a negative correlation was denoted by green edges. *Bifidobacterium* was indicated by the arrow. IBM = Infants fed with bacterial milk; ISM = Infants fed with sterile milk

Infants fed bacterial milk and infants fed sterile milk had similar intestinal microbial compositions, but microbial diversity varied. *Proteobacteria* and *Actinobacteria* constituted the dominant phylum in infant feces (Fig. 1b-c, Table 2). The most abundant genera were *Bifidobacterium*, *Escherichia-Shigella,* and *Bacteroides* (Fig. 1c). However, the IBM group had significantly higher Shannon and Simpson indices than the ISM group (*P* = 0.020, *P* = 0.048), revealing that infants fed bacterial milk have a richer microbial community (Fig. 2a-b). Beta diversity was estimated by using PCoA to seek interindividual differences in microbial communities. PCoA indicated similar clustering of microbiota among the IBM and ISM groups (*P* = 0.264, Fig. 2c).

We then drew a Venn diagram to visualize the shared and unique OTUs of the IBM and ISM groups. There were 273 shared OTUs between groups. Meanwhile, the IBM group has 1063 unique OTUs, which far exceeds those in the ISM group (Fig. 2d). This suggested that infants fed bacterial milk were more enriched with microbes.

The LEfSe analysis was performed to find biomarkers between the two groups. As illustrated in Fig. 2e, we found that *Eggerthellaceae, Lachnospiraceae, and Lachnospiraceae* were enriched in the IBM group. The abundance comparison diagram showed that *Eggerthellaceae* are absent from the ISM group, and *Lachnospiraceae* are only found in one infant gut (Fig. 2f-g).

Network analysis can reveal the internal relationships of a microbial community. We constructed the co-occurrence network and calculated the topological properties. The number of nodes, edges, and average degree in microbial network were higher in the IBM group (Fig. 2h-i, Table 4), which indicated the microbial community in infants fed with bacterial milk was more connected and complex.

**Table 4:** Topological properties of co-occurrence network

	IBM (*n* = 11)	ISM (*n* = 6)
Network diameter	8.0	3.0
Clustering coefficient	0.6	0.8
Average degree	6.2	2.5
Modularity	0.6	0.5
Nodes	44.0	25.0
Edges	136.0	31.0

Network diameter: The maximum distance between any two nodes in a network. Clustering coefficient: The percentage of neighbors of a node that can reach another neighbor without passing through it. Average degree: Edges of all nodes divided by total number of nodes. A network with a higher average degree is more complex. Modularity: A parameter that assesses whether the network can be divided into several modules. *IBM* Infants fed with bacterial milk, *ISM* Infants fed with sterile milk

### Microbial analysis of maternal gut microbiota

As there were no significant differences in clinical characteristics between pregnant women, the gut microbiota was further examined. The microbial composition of PBM and PSM groups was similar. The primary phyla were *Firmicutes* and *Bacteroidetes*, while the main genera were *Faecalibacterium* and *Bacteroides*. In addition, *Bifidobacterium* was more abundant in the PSM group (5.3%) than in the PBM group (1.5%) (Supplementary Table S2).

**Fig. 3:**
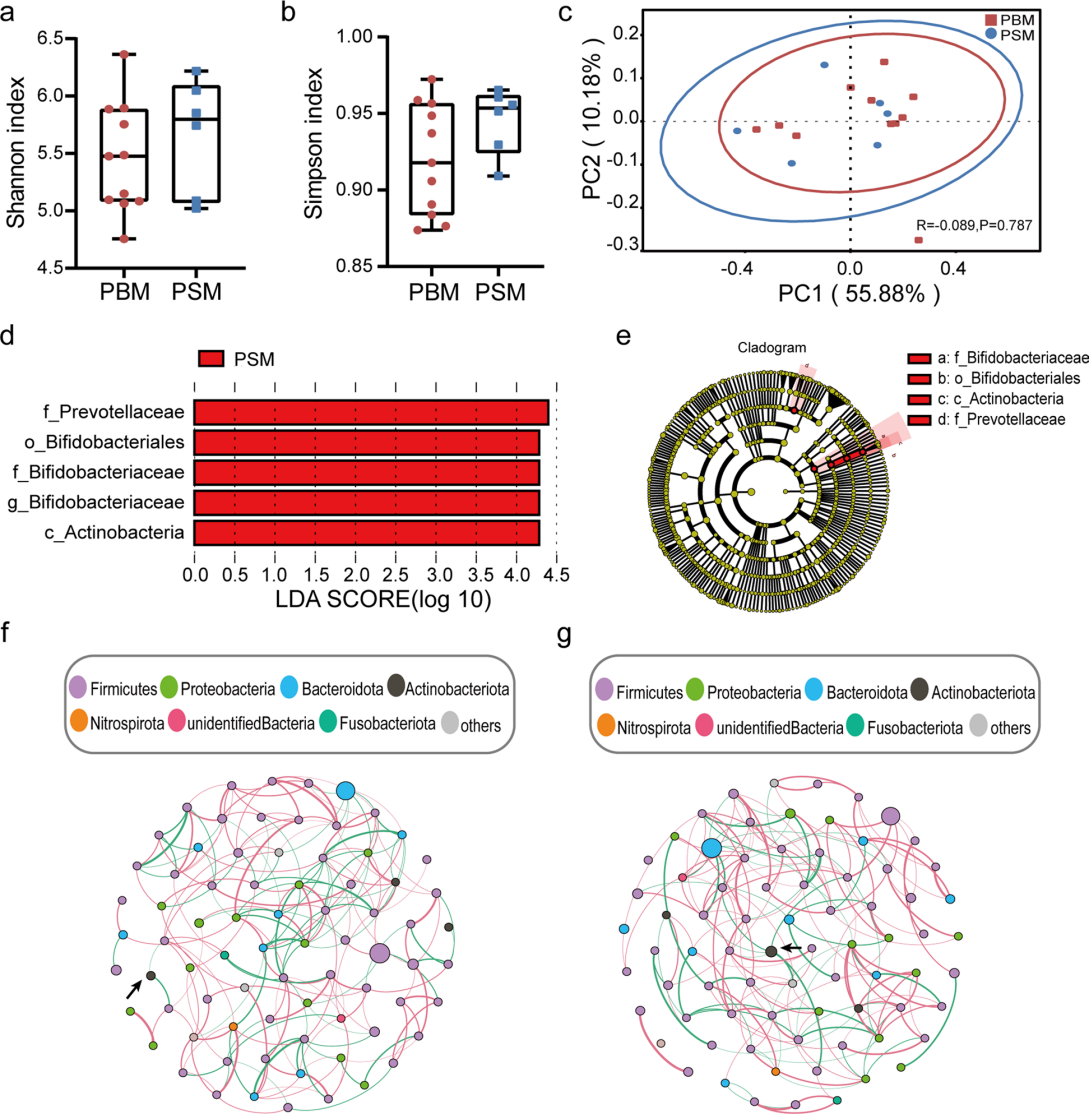
Microbial analysis of pregnant women’s fecal samples. Alpha diversity estimated by Shannon indices (**a**) and Simpson indices (**b**) showed no differences in groups. **c** PCoA based on weighted Unifrac distances indicated there were no differences in groups. Statistical significance was calculated by the Anosim test. **d** LEfSe analysis compared all taxonomy between groups. Identified five biomarkers when LDA score = 4. **e** Evolutionary branch diagram demonstrated that *Actinobacteria*, *Bifidobacteriales*, *Bifidobacteriaceae* and *Bifidobacterium* belong to the same branch. Co-occurrence networks of gut microbiota at the genus level were showen in (**f**) and (**g**). **f** Co-occurrence network of the PBM group. **g** Co-occurrence network of the PSM group. Nodes with relative abundance less than 0.005% were removed. The nodes were colored by phylum, and nodes’ size was proportional to the relative abundance of genera. Edges represented the relationships between the nodes. A positive correlation was shown by red edges, while a negative correlation was denoted by green edges. *Bifidobacterium* was indicated by the arrow. PBM = Pregnant women with bacterial milk; PSM = Pregnant women with sterile milk

To evaluate variations in the community structure and diversity between pregnant women with or without bacterial milk, we performed Alpha and Beta diversity. Shannon and Simpson indices revealed no significant differences between the PBM and PSM groups (*P* = 0.591, *P* = 0.180) (Fig. 3a-b). Meanwhile, PCoA showed no separation of communities between the PBM and PSM groups, suggesting that the two bacterial communities had similar compositions and structures (Fig. 3c).

To further search for specific taxa between the two groups, we performed a LEfSe analysis. A total of five biomarkers were identified when LDA score = 4. The PSM group had significantly more *Actinobacteria*, *Bifidobacteriales, Bifidobacteriaceae*, *Prevotellaceae*, and *Bifidobacterium* than the PBM group (Fig. 3d). The evolutionary branch diagram demonstrated that *Actinobacteria*, *Bifidobacteriales*, *Bifidobacteriaceae* and *Bifidobacterium* belong to the same branch (Fig. 3e). Co-occurrence networks were constructed to exhibit the relationship between microbes of pregnant women, focusing on the relationship between *Bifidobacterium* and other genera. It is worth noting that *Bifidobacterium* negatively correlated with six genera in the PSM group, while it negatively correlated with only one genus in the PBM group (Fig. 3f-g).

## Discussion

In the current study, we found some breast milk collected from healthy women does not contain any microbial community. We then analyzed the microbiota in breast milk and feces from infants and pregnant women. Our study showed that bacterial milk and sterile milk have different effects on infant gut microbiota. We did not prove that the gut microbiota of pregnant women affected the presence of microbes in milk. Nonetheless, as far as we know, this is the first report to compare intestinal microbiota in infants fed with bacterial milk and sterile milk.

### Healthy women milk microbial profile

In this study, microbes were detected in 11 of 17 breast milk samples, with a positive detection rate of 64.7%. This result was close to the detection rate of our previous study [6] and the study of Kordy et al. [7]. Many researchers have detected microorganisms in human breast milk. *Firmicutes* was reported to be the most abundant phylum in milk [27, 28]. The common genera in breast milk included *Staphylococcus*, *Streptococcus*, *Pseudomonas*, and *Lactobacillus* [20, 29–32]. In this study, *Firmicutes* were abundant in breast milk. Meanwhile, *Streptococcus* and *Staphylococcus* had the highest abundance, which was generally consistent with other studies [20, 27, 31, 32]. Furthermore, the milk was also rich in *Lactobacillus* in our study. However, the abundance of *Pseudomonas* in breast milk was low, and we detected the commensal skin bacterium *Propionibacterium* in only one milk sample. A previous research [19] comparing the breast milk bacterial communities of mothers from Spain, Finland, South Africa, and China, has discovered higher levels of *Pseudomonas* and *Propionibacterium* in the breast milk of Spanish mothers who delivered by cesarean section than those from other countries. Furthermore, *Pseudomonas* was much lower in Chinese women who delivered vaginally compared to those who delivered by cesarean section [19]. Therefore, we attribute differences in results to geographic location and delivery mode.

The detection rate of *Bifidobacterium* and *Bacteroides* in milk was as high as 90.9%. These bacteria are strict anaerobes that often reside in the gut and are unlikely to originate from the skin. A study has found the existence of gut-associated bacteria such as *Bifidobacterium* in maternal and infant feces and breast milk [25]. Moreover, Jost et al. [33] found a strain of *Bifidobacterium* in all three ecosystems of a mother-neonate pair. These findings indicate that some gut microbes can enter milk and then colonize neonatal intestinal.

### Milk microbiota may affect the formation of neonatal gut microbiota

It has been reported that delivery modes, feeding modes, gestational age at birth and timing of solid food introduction may affect bacterial communities in infant feces [1, 14, 15, 34]. Our study manifested that the presence or absence of milk microbes has a minor effect on infant gut microbial composition but has a greater impact on microbial diversity and microbial network relationships. *Proteobacteria* and *Actinobacteria* constituted the dominant phylum in infant feces, which was in accordance with Pannaraj’s report [1]. After being fed with bacterial milk, Alpha diversity and microbial network associations increased. By contrast, infants fed with sterile milk had fewer microbial species and lower microbial network complexity. Alpha diversity has been regarded as a marker of microbiome health, and a reduction in Alpha diversity is seen as a manifestation of intestinal malnutrition [35]. Low diversity is thought to be related to metabolic disorders [36]. In addition, it has been reported that microbial network stability is related to its complexity, and the complexity of the network will contribute to its stability [37]. *Lachnospiraceae,* which was abundant in the IBM group, is described as a potential beneficial bacteria [38]. *Lachnospiraceae* can generate butyrate by hydrolyzing amylase and sugar. Butyrate provides energy to colon cells and maintains the integrity of the intestinal barrier. It appears that bacterial milk may help infants shape a healthy gut microbiome. However, long-term follow up is required to track infants’ health outcomes.

Notably, the microbial composition of the IBM and ISM groups was similar, although some breast milk was not detected for bacteria. *Bifidobacterium* and *Bacteroides* were also enriched in the ISM group, which indicated that they could be transmitted to newborns in multiple ways. The researchers identified DNA and cellular structure of intestinal bacteria such as *Bifidobacterium* in the placenta, amniotic fluid, and fetal membrane [39–41], suggesting that the intestinal microbiota such as *Bifidobacterium* may have colonized the fetal intestine during pregnancy. In addition, prebiotics such as human milk oligosaccharides can promote the proliferation and growth of beneficial bacteria such as *Bifidobacterium* and *Lactobacillus* [16]. Therefore, we still recommend breastfeeding even if maternal milk contains extremely low bacteria or is even sterile. It is imperative to carefully consider whether microbiota interventions are necessary for infants fed with sterile milk.

### The reason why the bacteria were not detected in the milk is unclear

Geographical location, delivery mode, maternal BMI, infant gender and feeding mode could affect milk microbiota composition [20, 22, 27, 42, 43]. To figure out why some milk had very low bacterial content or was sterile, the participants’ characteristics were compared. The pregnant women all live in Guangzhou, China. Meanwhile, they delivered vaginally and fed their newborns exclusively with breast milk. There were no significant differences between the two groups in geographical location, delivery mode and feeding mode. Other clinical characteristics, such as age, BMI, gestational week and infant gender, showed no significant differences.

Since some researchers have proposed the entero-mammary pathway theory, we analyzed the gut microbiota of the PBM group and the PSM group. Gut microbiota composition and diversity were similar between the two groups. The main difference was that *Prevotellaceae* and *Bifidobacterium* were abundant in the PSM group, and *Bifidobacterium* had more negative correlations. Several in vitro and in vivo studies validated that *Bifidobacterium* was able to affect the rearrangement of tight junction proteins and strengthen the intestinal barrier [44–46]. We speculated that *Bifidobacterium* might inhibit bacterial growth and improve intestinal permeability, leading to a decrease in the migration of microbes entering milk via the entero-mammary pathway. In contrast, Simpson et al. investigated the effect of probiotic supplementation on maternal breast milk and reported that no significant changes occurred in milk microbial community despite maternal supplementation [10]. Of note, only a few samples could be sequenced in both the probiotic and placebo groups due to low bacterial DNA quantities [10]. It has been suggested that newborns’ oral cavity and breast skin may provide additional microbes for milk. Regrettably, we didn’t collect oral samples and skin samples. However, if there are multiple sources of milk microbes, there should be more bacteria in milk. Taken together, further research is needed to determine the cause of undetectable bacteria in milk.

### Limitations

Many factors affect milk and gut microbes, such as skin microbes, environmental factors, and diet. Although we attempted to control for differences in race, age, BMI, and delivery mode among participants, it cannot rule out the possibility that other factors influenced the study's results. Our sample size is small, and studies with a larger sample size are needed to reduce the bias. Nevertheless, this study found distinct differences in Alpha diversity and network associations among infants fed with bacterial milk and sterile milk. This result deserves to be of interest.

## Conclusions

Overall, some breast milk from healthy postpartum women failed to be sequenced due to low microbial DNA quantities or is sterile. Research is needed to explore the reasons for this phenomenon. Infants fed with bacterial milk had higher Alpha diversity and more complex microbiota networks. These findings provide novel insight into milk microbiota and infant gut microbiota.

## Supplementary Information

**Additional file 1:** **Table S1.** Relative abundance of human milk microbiota. **Table S2.** The relative abundance of the top 20 abundances at the genus level of the 5 groups.

## Data Availability

The datasets generated and/or analyzed during the current study are not publicly available but are available from the corresponding author on reasonable request.

## Abbreviations

OTUs: Operational taxonomic units
PCOA: Principal Coordinates Analysis
LEfSe: Linear discriminant analysis effect size
